# INTERSECTIONALITY OF SENSORY LOSS AND RACE: IMPLICATIONS FOR BRAIN AND MENTAL HEALTH IN OLDER ADULTS

**DOI:** 10.1093/geroni/igac059.943

**Published:** 2022-12-20

**Authors:** Jennifer Deal, Willa Brenowitz, Roland J Thorpe, Jr.

## Abstract

Despite recent evidence that sensory loss may increase risk of dementia, mood disorders, and poor physical health in older adults, nearly all research to-date has been conducted in populations that are predominantly White and from high-income countries. This session will investigate the intersectionality of race with sensory loss with a focus on health equity and inclusion of traditionally underrepresented populations. It will evaluate sensory loss across multiple senses, how patterns vary by race, and whether socioeconomic status can explain these differences. In the US, prevalence of impairment in vision, touch, and smell is lower in Whites compared to Black or Hispanics. Interestingly, hearing loss prevalence is lower in Blacks than Whites, but dementia incidence is higher. This session will describe incident dementia risk associated with hearing loss in Black compared to White Americans. It will also address the relationship between dual (hearing and vision) loss and depressive symptoms in India to explore the generalizability of findings to lower middle-income countries. Sensory impairments may intersect with racial discrimination to exacerbate social and health disparities. This session will also describe racial disparities in food insecurity among Americans with vision loss. Finally, we will present results of a randomized controlled trial of an innovative, community-delivered approach to affordable, accessible hearing care. The study represents the largest U.S.-based trial cohort to date of low-income older adults and older African American adults with hearing loss.

